# Broadband vectorial ultrathin optics with experimental efficiency up to 99% in the visible region via universal approximators

**DOI:** 10.1038/s41377-021-00489-7

**Published:** 2021-03-04

**Authors:** F. Getman, M. Makarenko, A. Burguete-Lopez, A. Fratalocchi

**Affiliations:** PRIMALIGHT, Faculty of Electrical Engineering; Applied Mathematics and Computational Science, King Abdullah University of Science and Technology, Thuwal, 23955-6900 Saudi Arabia

**Keywords:** Metamaterials, Optical materials and structures

## Abstract

Integrating conventional optics into compact nanostructured surfaces is the goal of flat optics. Despite the enormous progress in this technology, there are still critical challenges for real-world applications due to the limited operational efficiency in the visible region, on average lower than 60%, which originates from absorption losses in wavelength-thick (≈ 500 nm) structures. Another issue is the realization of on-demand optical components for controlling vectorial light at visible frequencies simultaneously in both reflection and transmission and with a predetermined wavefront shape. In this work, we developed an inverse design approach that allows the realization of highly efficient (up to 99%) ultrathin (down to 50 nm thick) optics for vectorial light control with broadband input–output responses in the visible and near-IR regions with a desired wavefront shape. The approach leverages suitably engineered semiconductor nanostructures, which behave as a neural network that can approximate a user-defined input–output function. Near-unity performance results from the ultrathin nature of these surfaces, which reduces absorption losses to near-negligible values. Experimentally, we discuss polarizing beam splitters, comparing their performance with the best results obtained from both direct and inverse design techniques, and new flat-optics components represented by dichroic mirrors and the basic unit of a flat-optics display that creates full colours by using only two subpixels, overcoming the limitations of conventional LCD/OLED technologies that require three subpixels for each composite colour. Our devices can be manufactured with a complementary metal-oxide-semiconductor (CMOS)-compatible process, making them scalable for mass production at low cost.

## Introduction

The production of lightweight and wearable optoelectronic devices is presently hampered by the bulky and expensive nature of traditional optical components^1–3^. Flat optics aims to address this problem by replacing conventional optics with highly integrated nanostructured surfaces. This technology has attracted enormous interest, with a large variety of designs demonstrated such as lenses^2,4–7^, holograms^8–12^, filters^13–15^ and other components capable of outperforming their traditional counterparts^16–23^.

In the visible range, the use of dielectric nanostructures for the production of these devices has gained favour, as metallic designs suffer from significant ohmic losses, particularly when transmissive elements are desired^24^. Currently, the challenges being addressed at visible frequencies are related to the scalability of the structure fabrication, the design of different types of broadband functionalities, and the increase of the operational and transmission efficiency, which is essential to enable complex layer-by-layer integration^25–27^. The majority of transmissive flat optics designed to operate in the visible region exploit propagation phase shifts in truncated waveguides^22^, using wavelength-thick structures in the range between 250 and 800 nm. While this approach works quite well at infrared frequencies, where commonly used dielectrics are highly transparent, in the visible region, it is severely limited by absorption losses. The best reported working efficiency of fabricated silicon devices, encompassing deflectors, holograms, lenses and polarization splitters, lies between 18% and 67%^10,28–30^. While devices based on TiO_2_, Si_3_N_4_ and GaN have shown promising results, reaching values as high as 91.6% for the focusing efficiency of a narrow-band metalens at 532 nm^31^, the lower refractive index of these materials necessitates the use of nanostructures with very high aspect ratios, often requiring manufacturing with atomic layer deposition, a method that is not scalable for mass production due to deposition rates of less than one angstrom per cycle^4,22,26,32–34^.

Another class of recently developed flat-optics devices exploits the interaction between suitably engineered electric and magnetic dipoles, reporting visible-range lenses and deflectors with measured efficiencies between 58% and 71%^30,35,36^. Electric and magnetic dipoles, however, allow the control of the system response within the bandwidth in which the dipoles overlap, which is typically a single frequency^37–40^.

The inverse design method has been recently investigated as an alternative to intuition-based flat optics design^41,42^. In this approach, the desired response is set as the input parameter, and the computer furnishes the material design by optimizing a figure of merit. Currently, approaches being explored are centred on the optimization of structures with periodic cells of assumed known period^43–46^ or on the generation of complex patterns from random refractive index distributions^25,47–50^. The majority of inverse design research is currently focused on the high-transparency region, at near-IR^47,48,51,52^ or gigahertz^53^ frequencies, with the best result being an up to 77% polarization efficiency for polarizing beam splitters. In the visible region, published inverse designs showed up to 67% focusing efficiency for metalenses^49^, reporting experimental performances comparable to the best available designs based on intuition.

A present limitation of inverse design is the lack of a universal strategy that is guaranteed to produce working devices with high efficiency: it is well known that optimization theory fails if the initial design is either too far from the solution or is developed along directions that are not convergent^54^.

A second important issue is that all design approaches (direct and inverse) proposed thus far at visible frequencies typically control the system response in either transmission or reflection, and do not yet address broadband vectorial light management simultaneously in both reflection and transmission and with a desired wavefront, such as in dichroic mirrors and other standard components that are not yet realized in flat optics. These points are particularly interesting with reference to the recent progress in diffractive deep optical networks at THz frequencies^55^ and neuromorphic computing with nonlinear waves^56^. If it could be possible to integrate some of these universal concepts into a flat optical structure, then we could engineer efficient, scalable and on-demand broadband optical components for light processing via flat surfaces.

The aim of this article is to explore a path to concurrently address the issues of efficiency, bandwidth, functional response and fabrication scalability. We begin by addressing the question of how wide can be the spectrum of functionalities that can be designed with a flat optical structure. We demonstrate that by suitably engineering semiconductor nanostructures, which behave as a neural network that can approximate a user-defined input–output function, it is possible to design flat optical surfaces that can represent arbitrarily defined output electromagnetic responses. We rigorously prove that the system possesses the same universal expressivity as a feedforward neural network with a nonpolynomial activation function and a variable threshold^57^. We then use this result to develop an inverse design approach along optimization lines that can be used to engineer structures with high efficiency that can be manufactured with mass-production-compatible techniques.

Universal approximators are enabled in a design strategy that controls by geometrical deformations a sufficiently large number of nanoscale resonances, theoretically equal to or larger than the number of design points in the frequency response of the flat-optics device. As this approach does not rely on propagation effects, it allows the thickness of the structure to be reduced to ultrathin values, as low as 50 nm, considerably reducing absorption losses at visible wavelengths. This approach also allows us to relax constraints on the shape. We mathematically demonstrate that these universal approximators can be generated in geometries that are as simple as possible, such as cuboid nanostructures, which can be manufactured by using a CMOS-compatible fabrication process scalable to mass production by nanoimprinting or deep UV^58^.

Implementing these results numerically requires performing a global optimization in a sufficiently large space of multimodal nanoresonators via first-principles simulations, allowing each nanostructure to explore all possible deformations, without making any assumption on the system periodicity. We address this issue by developing parallel software that couples large-scale optimization techniques with the latest generation of neural networks for computer vision^59^.

We validate these results by implementing a series of flat-optics devices for different applications, comparing their performance with both direct and inverse designed structures, and introducing new structures that are not yet realized with flat optics and are defined by wideband responses, ranging from 400 nm and spanning the entire visible range. In all these examples, we report experimentally measured efficiencies between 90% and 99% in the visible region for flat optical structures with thicknesses down to 50 nm for broadband vectorial light control simultaneously in both reflection and transmission, and with the desired wavefront shapes at visible wavelengths. These results show that improving the knowledge on light–matter interactions for strongly multimodal optical nanostructures helps in engineering high-performing nanomaterials.

## Results

### Theory of flat optics via universal approximators

**Fig. 1:**
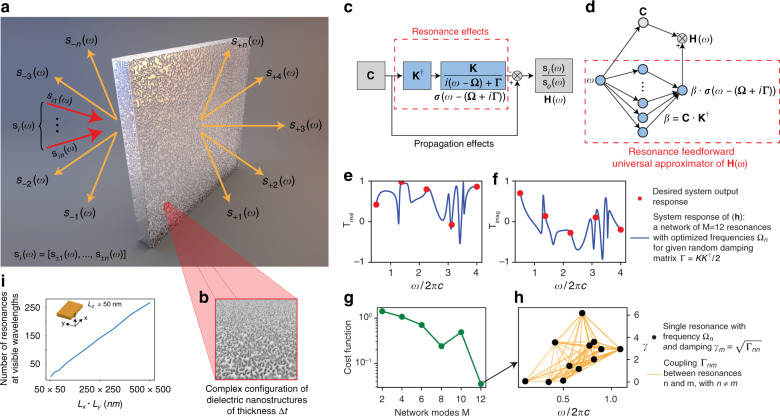
Universal flat-optics approximators: general idea. **a** Problem setup composed of a flat optical surface constituted by resonant nanostructures: **b** with input **s**_*i*_ and output **s**_*o*_ scattered waves. (**i**) Dependency of the number of resonances on the dimensions of a nanostructure. **c** Block diagram of the input–output transfer function ${\mathbf{H}}\left( \omega \right) = \frac{{{\mathbf{s}}_{\mathrm{o}}\left( \omega \right)}}{{{\mathbf{s}}_{\mathrm{i}}\left( \omega \right)}}$. **d** Equivalent representation of (**c**) with a feedforward single hidden layer neural network modelling the effects of the resonances. **e**–**h** Demonstration of the universal representation behaviour of (**d**): an arbitrarily defined system response (**e**, **f**, red markers) is obtained by tuning the resonances **Ω** of the network for given initial weights **β**, couplings **Κ** and damping **Γ**. The problem is solved by minimizing a cost function (**g**) by using an increasing number of resonances *M*, which defines the size of **Ω**. **h** Network configuration that represents the desired response (**e**, **f**, solid lines). The general demonstration of this result for an arbitrary structure is carried out in Supplementary Note II

Figure 1 summarizes the setup and main idea of this approach. An electromagnetic wave composed of a spectrum of waves with amplitudes $\left[ {s_{i1}\left( \omega \right), \ldots ,s_{in}\left( \omega \right)} \right] = {\mathbf{s}}_{\mathrm{i}}\left( \omega \right)$ (Fig. 1a, red arrows) impinges on an optical surface constituted by a complex distribution of dielectric nanostructures grown on a transparent substrate (Fig. 1b), generating both reflected *s*_*-n*_(*ω*) and transmitted *s*_*+n*_(*ω*) contributions propagating in different scattering directions (Fig. 1a, orange arrows). The system output response ${\mathbf{s}}_{\mathrm{o}}\left( \omega \right) = \left[ {s_{ \pm 1}\left( \omega \right), \ldots ,s_{ \pm n}\left( \omega \right)} \right]$ is composed of the vector containing all the scattered contributions emanating from the surface. The coefficients *s*_*ij*_ and *s*_*±n*_ represent the scalar amplitudes of impinging and scattered waves, respectively, which are represented, e.g. by planar waves. The main question we aim to answer is whether it is possible to design the surface of Fig. 1a to act as a universal approximator of arbitrarily defined input–output transfer functions of the form: ${\mathbf{H}}\left( \omega \right) = \frac{{{\mathbf{s}}_{\mathrm{o}}\left( \omega \right)}}{{{\mathbf{s}}_{\mathrm{i}}\left( \omega \right)}}$.

Figure 1c presents the input–output relationship of the system obtained from the generalized scattering theory, which provides an equivalent formulation of the Maxwell equations based on an intuitive division of the space into propagation and resonance effects^37,60,61^. A detailed demonstration of this result is presented in refs. ^60,61^, while here, we summarize the main aspects. In this representation, space is partitioned into two main sets: the resonant nanostructures (resonance space) and the remaining space composing the external environment (propagation space). Each nanostructure is seen from the outer space as an ideal perfect electric conductor (PEC) material possessing no resonance. The outer space is seen from each resonant nanostructure as a perfect magnetic conductor (PMC) material. With this representation, nanostructures are described by a set of $m = 1, \ldots ,M$ resonant modes of ideal PMC cavities, while the outer space is characterized by propagation effects of light scattered by an array of ideal PEC structures. The total output **s**_o_(*ω*) from the system, resulting from propagation and resonance effects, reads as:

1 $$\begin{array}{*{20}{c}} {{\mathbf{s}}_{\mathrm{o}}\left( \omega \right) = {\mathbf{C}} \cdot {\mathbf{s}}_{\mathrm{i}}\left( \omega \right) - {\mathbf{\beta}} \cdot {\mathbf{\sigma}} \left[ {\omega - \left( {{\mathbf{\Omega }} + i{\mathbf{\Gamma }}} \right)} \right] \cdot {\mathbf{s}}_{\mathrm{i}}\left( \omega \right)} \end{array}$$

where ${\mathbf{\sigma }}\left[ {\omega - \left( {{\mathbf{\Omega }} + i{\mathbf{\Gamma }}} \right)} \right] = \frac{{\mathbf{K}}}{{i\left( {\omega - {\mathbf{\Omega }}} \right) + {\mathbf{\Gamma }}}}$ is a diagonal matrix containing the resonant frequencies $\Omega _m = ({\mathbf{\Omega }})_{mm}$ of the $m = 1, \ldots ,M$ modes of the resonance space, ${\mathbf{\Gamma }} = \frac{{{\mathbf{KK}}^\dagger }}{2}$ is the matrix of damping coefficients, **Κ** accounts for the coupling between the resonance space and environment, ${\mathbf{\beta }} = {\mathbf{C}} \cdot {\mathbf{K}}^\dagger$, and **C** is a scattering matrix with ${\mathbf{C}}^\dagger {\mathbf{C}} = 1$. Equation (1) is composed of two main contributions: nonresonant ${\mathbf{C}} \cdot {\mathbf{s}}_{\mathrm{i}}\left( \omega \right)$ and resonant ${\mathbf{\beta }} \cdot {\mathbf{\sigma }}\left[ {\omega - \left( {{\mathbf{\Omega }} + i{\mathbf{\Gamma }}} \right)} \right] \cdot {\mathbf{s}}_{\mathrm{i}}\left( \omega \right)$ effects.

The resonance contribution (Fig. 1c, blue blocks) is equivalent to a feedforward neural network (Fig. 1d, network inside the dashed red box), which processes the spectral frequency *ω* at the input into the output ${\mathbf{\beta }} \cdot {\mathbf{\sigma }}\left[ {\omega - \left( {{\mathbf{\Omega }} + i{\mathbf{\Gamma }}} \right)} \right]$ through a hidden layer of resonant modes, which act as neural units with activation function **σ** and output weights **β** (Fig. 1d).

The activation ${\mathbf{\sigma }} = \frac{{\mathbf{K}}}{{i\left( {\omega - {\mathbf{\Omega }}} \right) + {\mathbf{\Gamma }}}}$ is a rational function (see Supplementary Note I) and satisfies the conditions for the universal approximation theorem of neural networks:^62^ the function is nonpolynomial and possesses the complex threshold parameters **Γ**–*i***Ω**. This implies that electromagnetic resonances can be used as universal approximators: Supplementary Note II rigorously demonstrates in the general case that by controlling the position of the *M* resonant frequencies in **Ω**, regardless of the values of **Γ**, **C** and **Κ**, it is possible to exactly set the output response **H**(*ω*) in amplitude and phase at $2L \le M - 1$ frequency points $\omega _l$ ($l = 1, \ldots ,L$) and, in the least-square sense, at a number of spectral points $2L \ge M$ in any desired scattering channel.

Figure 1e–h illustrates this point quantitatively. We considered an optical network initialized to random values of **Ω**, **Γ**, **C** and **Κ** and set the real and imaginary parts of the output transmission $s_{ + 1}^{\left( {{\mathrm{target}}} \right)} = T_{{\mathrm{real}}} + iT_{{\mathrm{imag}}}$ of one scattering channel to random values at *L* = 5 different spectral points (Fig. 1e, f, red markers). Initialization details are provided in Supplementary Note III. By using iterative optimization, we trained the resonant frequencies **Ω** to approximate the desired response by minimizing the cost function $F = \left| {\left| {s_{ + 1}^{\left( {{\mathrm{target}}} \right)} - s_{ + 1}^{\left( {{\mathrm{predicted}}} \right)}} \right|} \right|$, with $s_{ + 1}^{\left( {{\mathrm{predicted}}} \right)}$ being the output generated by the optical network with trained **Ω**. In the optimization procedure, we changed only the resonance frequencies (**Ω**)_*nn*_ without altering **Κ**, **Γ** or **C**. Figure 1g shows the value of the cost function for the best network obtained for increasing the number of modes *M*. Once the mode number becomes larger than 2*L* + 1, in agreement with the results of Supplementary Note II, the network represents the desired response (Fig. 1e, f, solid lines). Figure 1h visualizes the resonance network with *M* = 12, shown as a connected graph with nodes at $\left( {{{\Omega }}_n,{{\Gamma }}_{nn} \equiv \gamma _n} \right)$ and links between nodes *n* and *m* representing the mode-coupling strength $\left| {{{\Gamma }}_{nm}} \right|$.

By using universal approximators, the design of flat optics is reduced to learning a suitable set of resonant frequencies in the hidden layer of Fig. 1d. These frequencies are learned by geometrical deformations. As an example, for a cuboid optical dielectric resonator of refractive index *n*_*r*_ and dimensions *L*_*x*_*, L*_*y*_ and *L*_*z*_ terminated by PMC boundary conditions, the resonant frequencies Ω_*nmp*_ can be adjusted by deforming the resonator shape via *L*_*x*_*, L*_*y*_ and *L*_*z*_. Figure 1i presents the number of resonances at visible wavelengths between 300 and 800 nm contained in a silicon (Si) cuboid resonator of thickness *L*_*z*_ = 50 nm with variable dimensions *L*_*x*_ and *L*_*y*_, ranging from 50 to 500 nm. Subwavelength cuboid resonators contain hundreds of resonances, allowing the design of universal approximators for a broad range of desired frequency responses. Other shapes (e.g. cylindrical or spherical) provide similar results. In this work, we focus on cuboid shapes because of their fabrication versatility.

The main challenge in engineering Eq. (1) is the nonlinear relationship between the activation function $\sigma = \frac{{\mathbf{K}}}{{i\left( {\omega - {\mathbf{\Omega }}} \right) + {\mathbf{\Gamma }}}}$ and the resonant frequencies **Ω**: modifying the geometry of one resonator based on Eq. (1) modifies the response of all the others. This requires carrying out the search by simultaneously optimizing all the frequencies of the network. To perform this task, we developed Autonomous Learning Framework for Rule-based Evolutionary Design (ALFRED) software compatible with parallel supercomputer architectures.

**Fig. 2:**
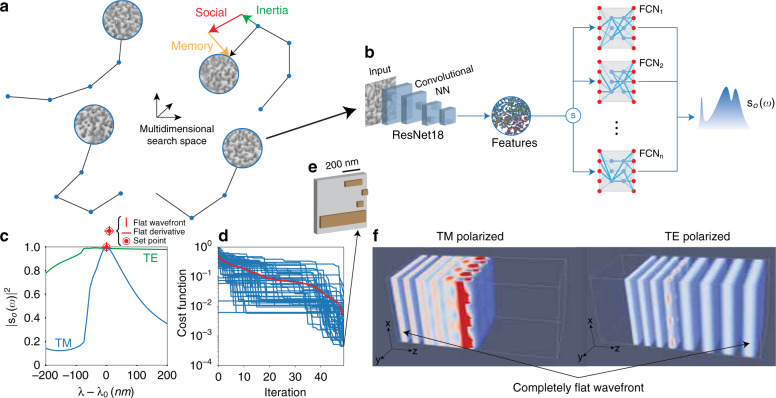
ALFRED design idea and application example. **a** Swarm parallel optimizer that searches the multidimensional space of solutions with an array of tentative geometries that use a cooperative scheme based on self and social interactions. **b** Predictor for each tentative solution: a convolutional neural network (CNN) extracts features from an input geometry and predicts the spectral response *s*_0_(*ω*) through a series of fully connected networks trained on different material thicknesses that are automatically selected by the switch (S). **c**–**f** Design example of a polarizing beam splitter. **c** Target response that maximizes the normal transmission for TE (green) and normal reflection for TM (blue) polarizations with a flat first-order derivative at the target design wavelength *λ*_0_ = 900 nm. **d** Cost function for progressive iterations of ALFRED (blue: particles, red: mean value). **e** Designed structure with a grey unit cell and brown nanoresonators. **f** FDTD simulations of (**e**) for TE and TM polarizations at *λ* = *λ*_0_

ALFRED consists of two main parts: an optimizer and a predictor (Fig. 2a). The global search for the best configuration of resonances is carried out by a particle swarm optimizer, which is effective in training high-dimensional neural networks^63,64^. The swarm performs a collective search based on an ensemble of randomly defined tentative particle solutions (Fig. 2a), with each particle representing a specific geometry of Si box resonators.

The swarm algorithm developed is a parallel version of that in refs. ^65,66^, in which the search parameters (inertia, social, and memory in Fig. 2a) are autonomously adapted from the collective interactions in the swarm. The cost function is defined as the norm between the desired response and the predicted response from the particle under consideration. In our implementation, the single particle evolution is carried out by a CPU core of a supercomputer architecture, and particle interactions are carried out in parallel between different cores, thus speeding up the global search.

The main bottleneck of the optimizer is the time required to evaluate the cost function: each particle needs to calculate the output response from the structure by using first-principles simulations, which are essential to take into account all material effects (e.g. dispersion) that can furnish a precise design. For this reason, we added a neural network predictor unit to each particle (Fig. 2b), which is trained by the finite-difference time-domain (FDTD) method to predict the outcome of first-principles simulations.

The predictor is designed with a convolutional neural network (CNN) based on the recently developed EfficientNet architecture^67^, which operates on an image representing a single cell of the structure, followed by a series of fully connected layers. The CNN extracts multidimensional features from the image and feeds them to the fully connected network (FCN) layer, which is trained to predict the output response **s**_o_(*ω*) from the features extracted by the CNN layer. We trained different FCNs with discrete thicknesses in the range from 50 to 300 nm with a step of 25 nm and connected them sequentially to the output from a CNN block through a logical switch (Fig. 2b, S), which chooses the appropriate block.

The training dataset for the predictor is composed of arrays of Si box structures (currently up to five) and is self-generated by ALFRED and autonomously optimized in the background. This is accomplished by mapping the dataset into a multidimensional feature space, generating additional datasets in the regions where the predictions are lower than a predetermined threshold (80%).

The results of Eq. (1) show that to reach the correct solution, the system has to be able to explore the space of all possible deformations of the resonator shape. To comply with this condition, ALFRED does not make any assumption on the system periodicity and optimizes this value autonomously.

Supplementary Fig. S1 shows a typical prediction example with the related dataset. The predictions match FDTD computations for both TE and TM input polarizations, with more than 99% of predictions above the threshold. During a typical search, ALFRED first uses the swarm equipped with the CNN + FCN predictor to rapidly converge to an initial structure, then removes the predictor and launches a final swarm optimization with parallel FDTD simulations to validate the exact design.

Figure 2c–f illustrates an example design obtained by ALFRED for a polarizing beam splitter at an operating wavelength of 900 nm. The minimized cost function *F* (Fig. 2c) is defined as:

2 $$\begin{array}{*{20}{c}} {F = \left| {\left| {1 - s_ + ^{{\mathrm{TE}}}\left( {\omega _0} \right)} \right|} \right| + \left| {\left| {1 - s_ - ^{{\mathrm{TM}}}\left( {\omega _0} \right)} \right|} \right| + \left| {\left| {\frac{{ds_ + ^{{\mathrm{TE}}}\left( {\omega _0} \right)}}{{d\omega }}} \right|} \right| + \left| {\left| {\frac{{ds_ - ^{{\mathrm{TM}}}\left( {\omega _0} \right)}}{{d\omega }}} \right|} \right|} \end{array}$$

where $s_ + ^{{\mathrm{TE}}}\left( {\omega _0} \right)$ and $s_ - ^{{\mathrm{TM}}}\left( {\omega _0} \right)$ are the transmission and reflection measured on a flat scattering wavefront at the operating frequency *ω*_0_ = 2*πc*/*λ*_0_ for TE and TM polarizations, respectively, and $\left| {\left| \cdot \right|} \right|$ is the norm. The cost function *F* maximizes the transmission for TE and the reflection for TM polarizations on a flat wavefront and provides broadband performances by minimizing the first derivative in transmission and reflection. Broader performances can be obtained by minimizing higher-order derivatives, which provides flatter frequency responses.

Figure 2d shows the values of the cost function minimized by ALFRED during progressive iterations. The final structure (Supplementary Fig. S1) is composed of four Si boxes of 100 nm thickness arranged in a complex geometrical pattern. The cost functions reach values below 10^−3^, which results in a device efficiency of 99.99%. Figure 2f illustrates this result quantitatively with FDTD simulations for the TE and TM polarizations at *ω*_0_, showing the behaviour of the structure with the generation of completely flat wavefronts in both reflection and transmission, without any wavefront aberration.

### Experiments: polarizing beam splitters, dichroic mirrors and two-subpixel colour displays

Using ALFRED, we designed, fabricated, and characterized a set of structures for different applications. Once the model was trained, numerical simulations were performed on a single GPU for a total simulation time of a few minutes. A strength of this technique is the use of the same manufacturing process (see Methods) for all devices based on the growth of amorphous Si on silica glass, thus minimizing the impact of different material surfaces on the final device performances.

We first considered a series of devices for polarization control in both transmission and reflection for a challenging component that is currently addressed by both direct and inverse design. This design is based on replicating the exact functionality of a bulk polarizing beam splitter, which either reflects at 180° or transmits with no deflection of light according to its TE or TM polarization. This functionality is different from that of the class of polarizing beam splitters intended to deflect an incident beam at different angles depending on its polarization and that have been previously reported in flat optics^68,69^.

We used ALFRED to design ultraflat polarizing beam splitters centred at different laser line frequencies, with full polarization control in reflection and transmission. The designs were obtained by minimizing the same fitness function of Eq. (2), illustrated in Fig. 2c, d, and by centring the wavelength *λ*_0_ around 533, 600 and 750 nm. In all cases, the results obtained are the same as in Fig. 2c, d: the fitness function is reduced to values below 10^−3^, corresponding to an efficiency over 99%, including material losses, for structures with a thicknesses of *Δt* = 50 nm (*λ*_0_ = 533 nm and *λ*_0_ = 750 nm) and *Δt* = 56 nm (*λ*_0_ = 600 nm), represented by different periodic configurations of Si boxes with periods Λ = 290 nm, Λ = 344 nm and Λ = 482 nm for the polarizers at *λ*_0_ = 533 nm, *λ*_0_ = 600 nm and *λ*_0_ = 750 nm, respectively. Figure 2e shows the geometry of a representative sample for *λ*_0_ = 900 nm.

**Fig. 3:**
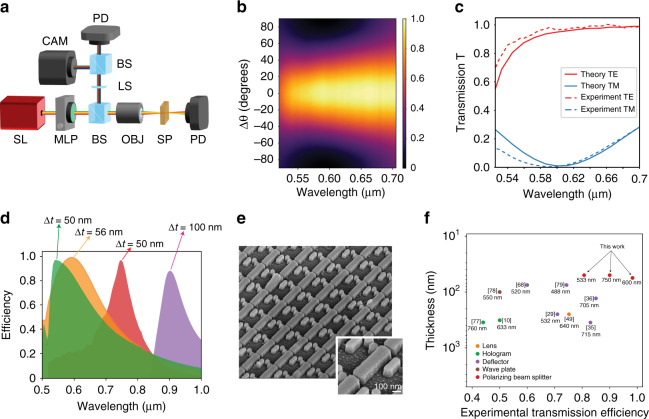
Experimental results for polarizing beam splitters. **a** Experimental setup used to characterize the samples (SL: supercontinuum laser, MLP: motorized linear polarizer, BS: beam splitter, LS: lens, OBJ: objective, CAM: CMOS camera, PD: calibrated photodetector, SP: sample). **b** Transmission efficiency of the 600 nm polarizer for different wavelengths and input polarization angles Δ*θ*. **c** Comparison between theory (solid line) and experiment (dashed line) for the 600 nm polarizer transmission curves. **d** Experimentally measured polarization efficiencies at different wavelengths for four different samples working around the common laser line wavelengths of 533, 600, 750 and 900 nm. **e** SEM images of a characteristic sample for the polarizer designed at 900 nm. **f** Silicon-based flat-optics state of the art in the visible range. The figure presents the experimentally measured transmission efficiency for previously reported devices in the visible region and for this work

We experimentally assessed the performances of 2 mm × 2 mm fabricated devices by the setup in Fig. 3a. We illuminated each sample with an NKT Photonics SuperK EXTREME supercontinuum laser source (LS, Fig. 3a), after the beam passed through a broadband linear polarizer mounted on a computer-controlled rotating stage (Fig. 3a, motorized linear polarizer, MLP), and then measured the transmission and reflection for each wavelength and each angular orientation Δ*θ* of the reference polarizer with two calibrated photodetectors (PD, Fig. 3a).

Figure 3b presents the transmission measurement result for the 600 nm polarizing beam splitter, showing a transmission beyond 95% over a large portion of the visible spectrum when aligned with the analyser and a rejection of over 95% of the light in a 75 nm range centred at the wavelength of 595 nm when perpendicular to the analyser. Figure 3c displays the theoretical (solid line) and experimental (dashed line) transmission spectra of the polarizer at 600 nm, illustrating good agreement between the designed and measured responses. Supplementary Fig. S2 shows the experimentally measured polarization behaviour at the 600 nm wavelength, exhibiting the expected Malus’s law sinusoidal curve^70^.

Figure 3d summarizes the polarization efficiency results measured for each sample and each wavelength, while Fig. 3e shows scanning electron microscope (SEM) images of a representative

sample. The polarization efficiency, defined as $\eta = \left( {\frac{{T_{{\mathrm{max}}} - T_{{\mathrm{min}}}}}{{T_{{\mathrm{max}}} + T_{{\mathrm{min}}}}}} \right)$^71^, is evaluated as *η* = 97%, *η* = 99% and *η* = 96% in the visible region for the 533, 600 and 750 nm polarizing beam splitters, respectively, and *η* = 88% in the near-IR region for the *λ*_0_ = 900 nm polarizer. Transmission/reflection flat-optics polarizers reaching polarization efficiencies of this high have only been reported in the near-IR around *λ* = 1500 nm^72,73^, where silicon has no losses. The approach presented in this work obtained similar results in the visible range at wavelengths at which Si is highly absorbing, proving to be a successful path to address the loss problem of high refractive index semiconductors in the design of highly efficient flat optics in the visible region.

To assess this statement quantitatively, we performed a detailed comparison with state-of-the-art visible flat optics, as shown in Fig. 3f, which presents the transmission efficiency of the best experimental realizations of silicon-based dielectric flat optics for the visible region. Our devices have experimental transmission efficiencies of 80.7%, 98.3% and 90% at their operating wavelengths of 533, 600 and 750 nm, respectively. Each design is shown to represent an improvement in terms of transmission efficiency compared to other devices operating at similar wavelengths. To the best of our knowledge, the polarizers reported here represent the first Si flat-optics devices showing experimental transmission efficiencies exceeding 90% in the visible region. These transmission values compare well with and in some cases even exceed those of commercially available polarizers, which typically range (see, e.g. the components available from Thorlabs or Newport) between 30% and 80% for 2 mm thick linear films and approximately 90% for 5–12 mm thick polarizing beam splitters.

**Fig. 4:**
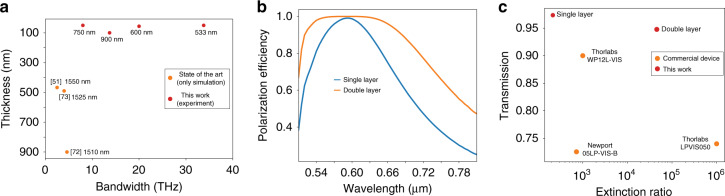
Comparison with state-of-the-art and cascaded systems. **a** Comparison of the fabricated polarizer bandwidths with the state-of-the-art bandwidths. **b** Polarization efficiency as a function of the number of cascaded polarizers. **c** Comparison of the fabricated polarizer transmission and extinction ratio with those of commercially available bulk polarizer components

Figure 4a presents a comparison of the polarizing bandwidth of our designs with those of the state-of-the-art flat-optics solutions currently proposed. To compare different devices, we used the common calculation of computing the operating bandwidth from the full-width at half-maximum (FWHM) of the extinction ratio curve. To the best of our knowledge, flat-optics polarizers in the visible range have not yet been experimentally implemented, and the closest solutions are represented by theoretical designs working at the telecommunication wavelength of 1550 nm. Notwithstanding these differences, the experimental bandwidth of the samples manufactured in this work significantly exceeds, by more than one order of magnitude, the operating bandwidth of the best theoretical design available in flat optics.

The performances of the polarizers can be enhanced with minimal losses by cascading multiple devices in series. Based on the previously described measurement results, Fig. 4b plots the experimental polarization efficiency for a single 600 nm polarizer and a two-layer configuration.

With two layers, the efficiency is maintained above 95% in a 130 nm range around the design wavelength while maintaining 95% transmission. Three layers could maintain near-unity polarization efficiency over the whole visible spectrum (300 nm) while maintaining 80% transmission efficiency, comparing well with the performances of broadband commercial polarizers from Thorlabs and Newport. Another approach to obtain a large bandwidth configuration is to employ ALFRED to design a single broadband polarizing layer using a broadband cost function with a target bandwidth of 200 nm (Supplementary Fig. S4).

Figure 4c presents a comparison of the fabricated 600 nm polarizing beam splitter and its associated, cascaded systems with commercially available polarizers. When cascaded, two layers achieve extinction ratios in the range of the commercial devices while exceeding their transmission performance without the need for anti-reflection coatings.

**Fig. 5:**
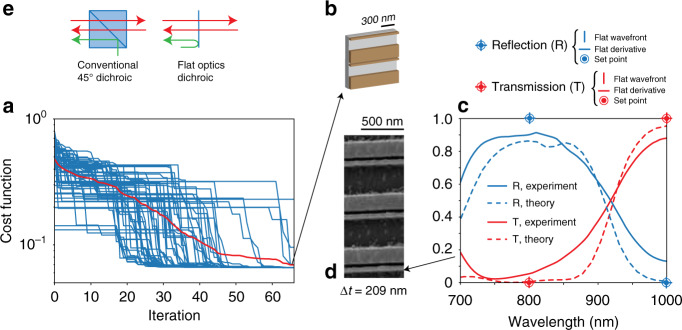
Flat-optics dichroic mirror. **a** Cost function of the target design for increasing iterations of ALFRED (blue: particles, red: mean value), and **b** sample design found. The dichroic component is designed based on 4 target points (**c**, blue/red markers), each characterized by a flat derivative and a flat wavefront. **c** Comparison of FDTD theoretical (dashed line) and experimental (solid line) results. **d** SEM image of a fabricated sample. **e** Conventional dichroic geometry, requiring 45° impinging light, versus the flat-optics structure working at normal incidence

Figure 5 depicts the design and characterization of a dichroic mirror, which acts as a wavelength demultiplexer by transmitting on-axis light at one frequency *ω*_0_ and reflecting on-axis light at a different frequency *ω*_1_≠*ω*_0_, maintaining a perfectly flat wavefront. To the best of our knowledge, there are no flat optics or bulk components that can perform this functionality. Additionally, we chose this example because it represents a broadband device with a response defined over hundreds of nm, representing an interesting broadband benchmark for ALFRED. The cost function for this device is defined as follows:

3 $$\begin{array}{*{20}{c}} {F = \left| {\left| {1 - s_ + ^{\mathrm{T}}\left( {\omega _0} \right)} \right|} \right| + \left| {\left| {1 - s_ - ^{\mathrm{R}}\left( {\omega _1} \right)} \right|} \right| + \left| {\left| {\frac{{ds_ + ^{\mathrm{T}}\left( {\omega _0} \right)}}{{d\omega }}} \right|} \right| + \left| {\left| {\frac{{ds_ - ^{\mathrm{R}}\left( {\omega _1} \right)}}{{d\omega }}} \right|} \right|} \end{array}$$

where $s_ + ^{\mathrm{T}}\left( \omega \right)$ and $s_ - ^{\mathrm{R}}\left( \omega \right)$ are the transmission and reflection of the structure measured on a flat scattering wavefront at frequency *ω*. Figure 5a presents the results of ALFRED for a swarm of 40 particles, showing a reduction in the cost function *F* to values below 10^−1^ after 60 iterations. The theoretical transmission on the flat front of the configuration found at 850 nm is 10^−2^ (i.e. 99.9% accuracy), and the transmission beyond 1000 nm is 0.98 (i.e. 98% accuracy). On the reflection side, the theoretical reflection on the flat front at 850 nm is 0.87 (i.e. 87% accuracy), and the reflection beyond 1000 nm is 10^−2^ (i.e. 99.9% accuracy). Both derivatives are almost zero, as requested of ALFRED. The design structure is composed of an aperiodic pattern of lines with thickness ∆*t* = 209 nm, period Λ = 542 nm, and widths 250 and 40 nm (Fig. 5b). These results also show the ability of ALFRED to explore a large manifold of solutions, converging automatically to diverse types of 2D/3D structures that can solve the problem with high efficiency.

Figure 5c (dashed lines) shows the FDTD-calculated spectral response of the structure, showing the required behaviour around the target points (Fig. 5b, circle markers) in a bandwidth larger than 250 nm, with transmission efficiencies above 95% and a reflection of less than 1%.

The experimental response of a fabricated sample (Fig. 5d) is presented in Fig. 5c (solid lines). The experimental results match the theory quite well in all of the frequency ranges considered, showing an experimental efficiency of 90% in transmission and a reflection in the range of a few percent, with differences of approximately 10% (Supplementary Fig. S3).

In contrast to a traditional dichroic mirror, which requires a macroscopic thickness due to the 45° wavelength mixing geometry (Fig. 5e), the flat-optics sample of Fig. 5d performs the same functionality at normal incidence and with a structure of only ≈200 nm thickness, allowing flat system integration.

**Fig. 6:**
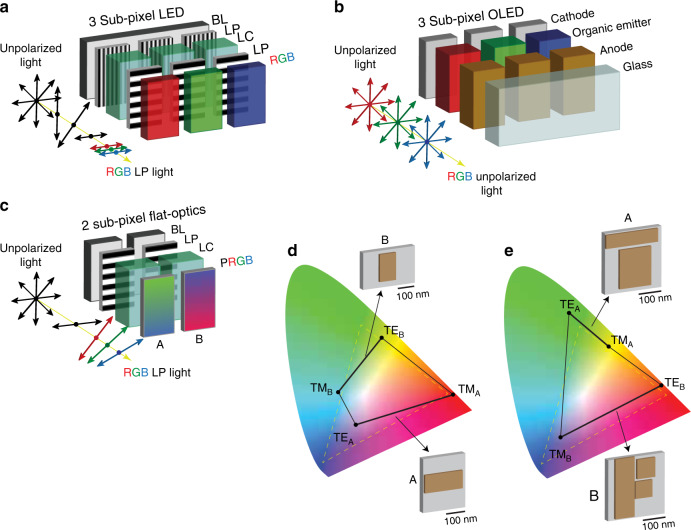
Two-subpixel flat-optics colour display: idea and design. **a, b** Basic working principle of current LCD/OLED colour technologies based on three red, green and blue (RGB) subpixel colour transmission units. **c** Proposed flat-optics technology based on two polarization-controlled subpixels A and B. **d, e** ALFRED designs of A and B (3D brown boxes with grey unit cells) with the chromaticity gamut contained in (**d**) and exceeding (**e**) the standard RGB colour space (dashed yellow line). In panels **d**, **e**, the chromaticity obtained when the input light varies between TE_*x*_ and TM_*x*_ polarizations for samples A and B is indicated as a solid thick black line, while the total gamut obtained by mixing the two subpixels is the four-point rhomboid delimited by the black lines

Figure 6 summarizes the design results for a backlit metasurface colour display based on two-subpixel technology, comparing it with the current state of the art. This example targets an inverse designed functionality defined over all of the visible bandwidth. In the current LCDs, colours result from unpolarized broadband backlight (BL, Fig. 6a), which is modulated in intensity via a liquid crystal cell (LC, Fig. 6a) equipped with two orthogonal linear polarizers (LP, Fig. 6a) and then filtered into primary red, green and blue components (RGB, Fig. 6a). In displays based on organic LEDs, colours are directly produced by organic monochromatic emitters at different frequencies, which are independently controlled (Fig. 6b). In both displays, composite colours are obtained by controlling the intensity of the three primary components.

This technology is inherently different from structural colouration^15,74^, in which a single structure is designed to diffract out a single colour, with different colours generated by engineering different structures. A flat-optics display requires engineering a single flat-optics device that simultaneously presents the full gamut of colours with no change in the device structure. To the best of our knowledge, no flat-optics structure has been previously designed to address this problem.

Here, we designed an integrated architecture (Fig. 6c) that allows polarization-intensity gamut control with only two subpixels. The unit cell uses two independently controlled backlight sources, followed by a polarization stage composed, as in LCDs, of a linear polarizer and a liquid crystal cell (LP + LC, Fig. 6c), in which the output light polarization state is rotated. The final colours are produced by employing two flat-optics polarization filters A and B, which output different chromaticities when the input light polarization changes orientation. To the best of our knowledge, no bulk photonics component exists that can perform this functionality.

**Fig. 7:**
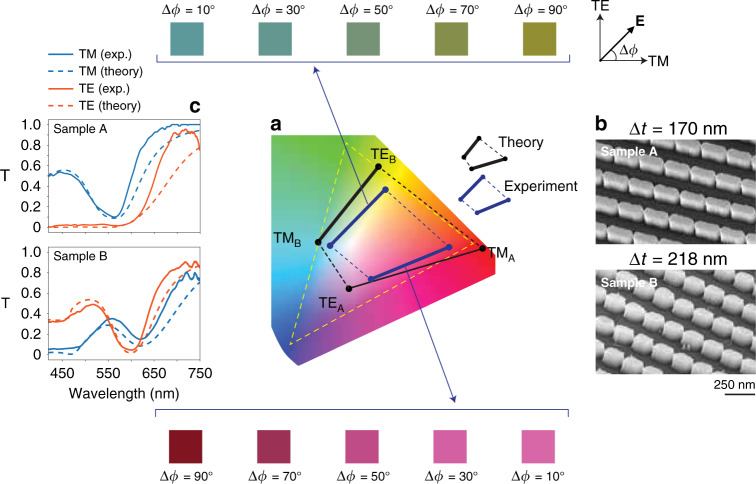
Characterization of the fabricated two-subpixel colour samples. **a** Theoretical (black rhomboid) and experimental (blue rhomboid) chromaticity gamut of samples A and B illustrated in the SEM images of panel (**b**). The corresponding colours for different input polarization angles Δ*ϕ* are shown in panel (**a**). **c** FDTD-calculated theoretical transmission (*T*) response (dashed line) compared to experimental measurements (solid line)

By generating RGB composite colours from two subpixels, this architecture allows the energy consumption of a traditional screen to be reduced by 33%, and for the same screen size, it allows an increase in the resolution by 33%. To design the required structures, we maximized the following cost function *F*:

4 $$\begin{array}{*{20}{c}} {F = {\mathrm{Area}}_{{\mathrm{gamut}}}\left[ {{\mathrm{A}}_{{\mathrm{pol,int}}},{\mathrm{B}}_{{\mathrm{pol,int}}}} \right]} \end{array}$$

representing the gamut area of all the possible chromaticities obtained by combining different polarizations and intensities at the input of samples A and B. To perform this calculation, we convert the transmitted electromagnetic spectra for samples A and B illuminated by TE/TM polarizations into *xy* chromaticity coordinates of a standard CIE 1931 chromaticity diagram^75^. The gamut of all possible chromaticities is the area of the rhomboid whose four vertices are the *xy* coordinates TE_A_, TM_A_, TE_B_ and TM_B_ of the spectra obtained from the two samples, A and B, under TE and TM polarization. This results from the condition that the combination in intensity of two chromaticity coordinates generates all the possible points on the line connecting the two initial coordinates^76–79^. Figure 6d, e illustrates the results of two sets of optimal structures found by ALFRED. The first design is composed of A and B samples constituted by periodic cells containing one box each (Fig. 6d, grey/brown unit cells), with thicknesses ∆*t* = 170 nm and ∆*t* = 218 nm, respectively. When the polarization changes from TE to TM, sample A presents blue-red colours (Fig. 6d, TE_A_–TM_A_ line), while sample B generates green to blue (Fig. 6d, TE_B_–TM_B_ line). The second sample configuration (Fig. 6e) is composed of unit cells with complex configurations of two and three boxes possessing a thickness of Δ*t* = 225 nm. This set of samples provides an improved performance, exhibiting a larger colour gamut that even exceeds the standard RGB spectrum (Fig. 6d, dashed yellow line).We demonstrate the proof of concept of this technology by manufacturing the optimized structure in Fig. 6d, whose chromaticity gamut lies mostly within the RGB spectrum and can be visualized with a standard RGB camera. We characterized the performance of the samples by using the setup in Fig. 3a, in which the MLP stage mimics the role of the liquid crystal cell, using the illumination of a white LED source in place of the supercontinuum laser. Figure 7a compares the theoretical (black rhomboid) and experimental (blue rhomboid) gamut curves obtained from manufactured samples A and B (Fig. 7b). Different colours are reported for each sample and for varying polarization angle ∆*φ* of the electric field **E**, varied between 0° and 90°, whose extrema represent TM and TE polarization, respectively. Figure 7c presents the corresponding measured spectra (solid lines) compared to the theoretical predictions from ALFRED (solid lines). The experimental results match the theory with good agreement, both in the spectral response (Fig. 7c) and in the experimentally retrieved colours (Fig. 7a), with average differences of a few percent between the wavelengths of 300 and 650 nm and between 10% and 15% beyond 650 nm. The latter differences in the measured spectra for wavelengths larger than 600 nm in Fig. 7c generate the small deviations observed in Fig. 7a in the blue and black rhomboids. Figure 7c shows that both samples A and B have almost ideal, near-unity, maximum transmission values.

### Controlling fabrication tolerances

Fabrication processes inevitably introduce manufacturing errors, and realized devices can behave differently from target devices. While this problem is almost impossible to address via direct intuition, an artificial intelligence inverse design strategy can provide a systematic approach to address this problem. To this end, we enhance the definition of the cost function in ALFRED as follows. We denote the original cost function discussed above (Eq. (2)) and fed from a nanoresonator geometry as *F*_geom_, with ‘geom’ being the parameters that define the nanoresonator structure. Starting from this cost function, we define a new generalized cost function that ALFRED is required to minimize as *F*_g_(geom). The new cost function *F*_g_(geom) considers $n = 1, \ldots ,N$ additional geometries ${\mathrm{geom}_{1}},{\mathrm{geom}_{2}}, \ldots ,{\mathrm{geom}}_{N}$, which are obtained by random perturbation of the original geometry ‘geom’. We evaluate the differences via a weighted mean square error with weight coefficient *α*:

5 $$\begin{array}{*{20}{c}} {{\mathrm{F}}_{\mathrm{g}}\left( {{\mathrm{geom}}} \right) = F\left( {{\mathrm{geom}}} \right) + \alpha \sqrt {\frac{1}{n}\mathop {\sum }\limits_{i = 1}^n \left( {F\left( {{\mathrm{geom}}_{i}} \right) - F\left( {{\mathrm{geom}}} \right)} \right)^2} } \end{array}$$

Minimizing *F*_g_(geom) implies finding the best solution that represents the required target points *F*(geom) (functionality) and that also minimizes the differences with respect to other geometries obtained via random perturbations mimicking fabrication errors (robustness). In this representation, the coefficient *α* is an additional degree of freedom that allows the user to select the weight of the robustness part versus the functionality part. Here, we use *α* = 0.5.

To illustrate the application of this idea, we designed a polarizer operating at 800 nm with *α* = 0 (i.e. no robustness) and *α* = 0.5 (robustness to fabrication errors). Details on the simulation parameters are provided in Supplementary Note IV. The results are summarized in Supplementary Fig. S5.

Supplementary Fig. S5a–c shows the results for the case of *α* = 0: while the performance of the default design (solid red line in Supplementary Fig. S5c) is well within the target, devices obtained under perturbations (dotted line) can present sensible differences according to the random perturbation, with polarization efficiencies as low as 20% in some cases. In contrast, the performances of the robust design (Supplementary Fig. S5d–f) are significantly better: the majority of designs stay within 90% of the target performance (Supplementary Fig. 5d).

The approach described by the generalized cost function Eq. (5) is general and can be applied to any flat-optics design created via ALFRED.

## Discussion

The designs presented were implemented by using the same industrially scalable manufacturing process based on Si. While this material is chosen due to its compatibility with CMOS mass production at inexpensive costs, the results of this work also apply to other semiconductors, including GaN, GaAs and Ge. The problem of absorption in the visible region is handled by inverse designing ultrathin nanoresonator structures, which render the device transparent across all of the visible range.

The key to enabling these designs lies in the theoretical demonstration that dielectric nanoresonators can act as universal approximators, which can in principle be used to engineer any possible optical response. This technology simultaneously controls the device transmission, reflection and desired output wavefront shape, with no distortion, and does not employ predefined assumptions on the system periodicity, converging automatically to globally optimized solutions characterized by high efficiency values over large design bandwidths. This approach is very versatile and can be easily generalized to include design robustness against manufacturing errors.

While the devices presented above show high working efficiency, with performances exceeding those of current commercial devices from both Thorlabs and Newport, they do not represent the best possible results that can be achieved with this approach. The optimization process we used was constrained to work with arrays of silicon boxes in an effort to ensure that the designs could be fabricated, limiting the search space in which ALFRED was trained. In addition, the training of the neural network architecture was limited, due to the computational resources, to less than 10 nanoresonators. These areas represent opportunities to be addressed in future works. Another interesting point is the role of nonlinearity. In this work, we limited our research to linear devices. However, the availability of active materials with a high refractive index and nonlinear (tuneable) emission in the visible region, such as GaN, represents a possible avenue of research for investigating nonlinear flat optics, such as wavelength converters and other nonlinear devices^40^. We hope that these results open the door for the realization of robust, integrated surfaces for linear and nonlinear light processing with high efficiency at visible frequencies and of integrated optics in ultrathin flat surfaces with efficiency comparable to their traditional bulk counterparts.

## Materials and methods

### Sample nanofabrication

We used, as a base wafer, a square piece of borosilicate glass, 18 mm wide and approximately 200 μm thick (12–540-A, from Fisher Scientific), which was cleaned by acetone and isopropyl alcohol. We then grew a uniform layer of amorphous silicon via plasma-enhanced chemical vapour deposition (PECVD). We controlled the thickness of the Si layer by using ellipsometry (UVISEL Plus, from HORIBA). We then spin coated the positive electron beam resist ZEP 520A (from ZEON Corporation) on the sample at 4000 RPM for 60 s, after which we baked it on a hotplate at 180 °C for 3 min. After this step, we spin coated the conductive polymer AR-PC 5090.02 (ALLRESIST) onto the sample at 4000 RPM for 60 s and baked the device again on a hotplate for 1 min at 100 °C. We wrote the optical resonator pattern by using a JEOL JBX-6300FS electron beam lithography system at an accelerating voltage of 100 kV. After writing, we removed the polymer by submerging the sample in deionized water for 60 s and developed the resist with n-Amyl acetate (ZED-N50 from ZEON Corporation) for 90 s and by submersion in isopropyl alcohol for 90 s. We then used electron beam evaporation to deposit a 22 nm layer of chromium on the sample. We performed lift-off by submerging the sample in *N*-methyl-2-pyrrolidone (ALLRESIST) at 70 °C for 1 h and sonicated the solution for one minute afterwards to create a protective mask in the image of the resonator pattern that we intended to fabricate. We then used reactive ion etching with SF_6_ to remove the unprotected silicon and expose the underlying glass. We removed the chromium mask by submersion in a perchloric acid and ceric ammonium nitrate solution (TechniEtch Cr01 from MicroChemicals) for 30 s.

## Supplementary information

Supplementary Information for: Broadband vectorial ultrathin optics with experimental efficiency up to 99% in the visible region via universal approximators.

## Data Availability

A version of the inverse design software ALFRED is available at https://github.com/makamoa/alfred. Instructions, data for training, and licensing terms are included in that repository.

## References

[1] Yu NF, Capasso F (2014). Flat optics with designer metasurfaces. Nat. Mater..

[2] Chen WT (2018). A broadband achromatic metalens for focusing and imaging in the visible. Nat. Nanotechnol..

[3] He Q (2018). High-efficiency metasurfaces: principles, realizations, and applications. Adv. Optical Mater..

[4] Khorasaninejad M (2016). Metalenses at visible wavelengths: diffraction-limited focusing and subwavelength resolution imaging. Science.

[5] Zheng GX (2017). Dual field-of-view step-zoom metalens. Opt. Lett..

[6] Shrestha S (2018). Broadband achromatic dielectric metalenses. Light Sci. Appl..

[7] Wang SM (2018). A broadband achromatic metalens in the visible. Nat. Nanotechnol..

[8] Overvig AC (2019). Dielectric metasurfaces for complete and independent control of the optical amplitude and phase. Light Sci. Appl..

[9] Chong KE (2016). Efficient polarization-insensitive complex wavefront control using Huygens’ metasurfaces based on dielectric resonant meta-atoms. ACS Photonics.

[10] Wang B (2016). Visible-frequency dielectric metasurfaces for multiwavelength achromatic and highly dispersive holograms. Nano Lett..

[11] Ren HR (2019). Metasurface orbital angular momentum holography. Nat. Commun..

[12] Huang LL, Zhang S, Zentgraf T (2018). Metasurface holography: from fundamentals to applications. Nanophotonics.

[13] Wood T (2017). All-dielectric color filters using SiGe-based Mie resonator arrays. ACS Photonics.

[14] Bonifazi M (2020). Free-electron transparent metasurfaces with controllable losses for broadband light manipulation with nanometer resolution. Adv. Optical Mater..

[15] Galinski H (2017). Scalable, ultra-resistant structural colors based on network metamaterials. Light Sci. Appl..

[16] Decker M, Staude I (2016). Resonant dielectric nanostructures: a low-loss platform for functional nanophotonics. J. Opt..

[17] Staude I, Schilling J (2017). Metamaterial-inspired silicon nanophotonics. Nat. Photonics.

[18] Chang SY, Guo XX, Ni XJ (2018). Optical metasurfaces: progress and applications. Annu. Rev. Mater. Res..

[19] Yang ZJ (2017). Dielectric nanoresonators for light manipulation. Phys. Rep..

[20] Kuznetsov AI (2016). Optically resonant dielectric nanostructures. Science.

[21] Jahani S, Jacob Z (2016). All-dielectric metamaterials. Nat. Nanotechnol..

[22] Colburn S (2018). Broadband transparent and CMOS-compatible flat optics with silicon nitride metasurfaces [Invited]. Optical Mater. Express.

[23] Glybovski SB (2016). Metasurfaces: from microwaves to visible. Phys. Rep..

[24] Shalaev MI (2015). High-efficiency all-dielectric metasurfaces for ultracompact beam manipulation in transmission mode. Nano Lett..

[25] Liu ZC (2018). Generative model for the inverse design of metasurfaces. Nano Lett..

[26] Chen WT (2019). A broadband achromatic polarization-insensitive metalens consisting of anisotropic nanostructures. Nat. Commun..

[27] Shibanuma T, Albella P, Maier SA (2016). Unidirectional light scattering with high efficiency at optical frequencies based on low-loss dielectric nanoantennas. Nanoscale.

[28] Li J (2019). Efficient polarization beam splitter based on all-dielectric metasurface in visible region. Nanoscale Res. Lett..

[29] Zhou ZP (2017). Efficient silicon metasurfaces for visible light. ACS Photonics.

[30] Cheng JR, Jafar-Zanjani S, Mosallaei H (2016). All-dielectric ultrathin conformal metasurfaces: lensing and cloaking applications at 532 nm wavelength. Sci. Rep..

[31] Chen BH (2017). GaN metalens for pixel-level full-color routing at visible light. Nano Lett..

[32] Devlin RC (2016). Broadband high-efficiency dielectric metasurfaces for the visible spectrum. Proc. Natl Acad. Sci. USA.

[33] Mueller JPB (2017). Metasurface polarization optics: independent phase control of arbitrary orthogonal states of polarization. Phys. Rev. Lett..

[34] Johnson RW, Hultqvist A, Bent SF (2014). A brief review of atomic layer deposition: from fundamentals to applications. Mater. Today.

[35] Aoni RA (2019). High-efficiency visible light manipulation using dielectric metasurfaces. Sci. Rep..

[36] Yu YF (2015). High-transmission dielectric metasurface with 2*π* phase control at visible wavelengths. Laser Photonics Rev..

[37] Koshelev K (2019). Nonradiating photonics with resonant dielectric nanostructures. Nanophotonics.

[38] Gongora JST (2017). Anapole nanolasers for mode-locking and ultrafast pulse generation. Nat. Commun..

[39] Ollanik AJ (2018). High-efficiency all-dielectric huygens metasurfaces from the ultraviolet to the infrared. ACS Photonics.

[40] Kivshar Y (2018). All-dielectric meta-optics and nonlinear nanophotonics. Natl Sci. Rev..

[41] Pilozzi L (2018). Machine learning inverse problem for topological photonics. Commun. Phys..

[42] Molesky S (2018). Inverse design in nanophotonics. Nat. Photonics.

[43] Malkiel I (2018). Plasmonic nanostructure design and characterization via deep learning. Light Sci. Appl..

[44] Lin RH (2020). Inverse design of plasmonic metasurfaces by convolutional neural network. Opt. Lett..

[45] Nadell CC (2019). Deep learning for accelerated all-dielectric metasurface design. Opt. Express.

[46] Liu DJ (2018). Training deep neural networks for the inverse design of nanophotonic structures. ACS Photonics.

[47] Sell D (2017). Large-angle, multifunctional metagratings based on freeform multimode geometries. Nano Lett..

[48] Sell D (2017). Periodic dielectric metasurfaces with high-efficiency, multiwavelength functionalities. Adv. Optical Mater..

[49] Phan T (2019). High-efficiency, large-area, topology-optimized metasurfaces. Light Sci. Appl..

[50] Andkjær J (2014). Inverse design of nanostructured surfaces for color effects. J. Optical Soc. Am. B.

[51] Shen B (2014). Ultra-high-efficiency metamaterial polarizer. Optica.

[52] Singh, R. et al. Inverse design of photonic metasurface gratings for beam collimation in opto-fluidic sensing. Preprint at: arXiv Prepr. arXiv 1911.08957 (2019).

[53] Callewaert F (2018). Inverse-designed broadband all-dielectric electromagnetic metadevices. Sci. Rep..

[54] Gendreau, M. & Potvin, J. Y. *Handbook of Metaheuristics* (Springer, 2019).

[55] Lin X (2018). All-optical machine learning using diffractive deep neural networks. Science.

[56] Marcucci, G., Pierangeli, D. & Conti, C. Theory of neuromorphic computing by waves: machine learning by rogue waves, dispersive shocks, and solitons. *Phys. Rev. Lett.* **125**, 093901 (2020). 10.1103/PhysRevLett.125.093901 32915624

[57] Haykin SS (2009). Neural Networks and Learning Machines..

[58] Guo LJ (2007). Nanoimprint lithography: methods and material requirements. Adv. Mater..

[59] Burguete-Lopez, A. et al. Broadband ultra-flat optics with experimental efficiencies exceeding 99% at visible wavelengths. In *14*^*th*^*Pacific Rim Conference on Lasers and Electro-Optics (CLEO PR 2020)* (OSA, 2020).

[60] Gongora JST, Favraud G, Fratalocchi A (2017). Fundamental and high-order anapoles in all-dielectric metamaterials via Fano–Feshbach modes competition. Nanotechnology.

[61] Makarenko M (2020). Generalized Maxwell projections for multi-mode network Photonics. Sci. Rep..

[62] Leshno M (1993). Multilayer feedforward networks with a nonpolynomial activation function can approximate any function. Neural Netw..

[63] Kiranyaz, S., Ince, T. & Gabbouj, M. *Multidimensional Particle Swarm Optimization for Machine Learning and Pattern Recognition* (Springer, 2014).

[64] Kennedy, J. F., Eberhart, R. C. & Shi, Y. H. *Swarm Intelligence* (Morgan Kaufmann Publishers, 2001).

[65] Iadevaia S (2010). Identification of optimal drug combinations targeting cellular networks: integrating phospho-proteomics and computational network analysis. Cancer Res..

[66] Liu, M. S., Shin, D. & Kang, H. I. Parameter estimation in dynamic biochemical systems based on adaptive particle swarm optimization. In *Proc. 2009 7th International Conference on Information, Communications and Signal Processing*. 1–5 (IEEE, 2009).

[67] Tan, M. X. & Le, Q. V. EfficientNet: rethinking model scaling for convolutional neural networks. In *Proc. 36th International Conference on Machine Learning*, *PMLR* (eds. Chaudhuri, K. & Salakhutdinov, R.) Vol. 97, 6105–6114 (PMLR, 2019).

[68] Lin DM (2017). Optical metasurfaces for high angle steering at visible wavelengths. Sci. Rep..

[69] Khorasaninejad M, Zhu W, Crozier KB (2015). Efficient polarization beam splitter pixels based on a dielectric metasurface. Optica.

[70] Collett, E. *Field Guide to Polarization* (SPIE Press, 2005).

[71] Bass, M*. Handbook of Optics. Geometrical and Physical Optics, Polarized Light, Components and Instruments.* 3rd edn., Vol. I (McGraw-Hill, 2010).

[72] Wu HM (2009). Polarizing beam splitter based on a subwavelength asymmetric profile grating. J. Opt..

[73] Zheng GX (2016). Ultracompact high-efficiency polarising beam splitter based on silicon nanobrick arrays. Opt. Express.

[74] Yang B (2018). Polarization-sensitive structural colors with hue-and-saturation tuning based on all-dielectric nanopixels. Adv. Optical Mater..

[75] Luo MR (2016). Encyclopedia of Color Science and Technology.

[76] Billmeyer FWJr (1983). Color science: concepts and methods, quantitative data and formulae, 2nd ed., by Gunter Wyszecki and W. S. Stiles, John Wiley and Sons, New York, 1982, 950pp. Price: $75.00.. Color Res. Appl..

[77] Li QT (2016). Polarization-independent and high-efficiency dielectric metasurfaces for visible light. Opt. Express.

[78] Lin DM (2014). Dielectric gradient metasurface optical elements. Science.

[79] Sell D (2016). Visible light metasurfaces based on single-crystal silicon. ACS Photonics.

