# Supplementary material for: Broadband vectorial ultrathin optics with experimental efficiency up to 99% in the visible region via universal approximators
Source: Light Sci Appl. 2021 Mar 4;10:47. doi: 10.1038/s41377-021-00489-7 (PMC7977065; doi:10.1038/s41377-021-00489-7)
Supplement: Supplementary file 1 — Supplementary Information for: Broadband vectorial ultrathin optics with experimental efficiency up to 99% in the visible region via universal approximators. [file 41377_2021_489_MOESM1_ESM.pdf]

Supplementary Information for: Broadband vectorial ultrathin optics with experimental efficiency up to 99% in the visible region via universal approximators

F. Getman[1†], M. Makarenko[1†], A. Burguete-Lopez[1†] & A. Fratalocchi[1*]
[1] *PRIMALIGHT, Faculty of Electrical Engineering; Applied Mathematics and Computational Science, King Abdullah University of Science and Technology, Thuwal 23955-6900, Saudi Arabia*
[†] *These authors contributed equally to this work.*
Contact details:
F. Getman - fedor.getman@kaust.edu.sa,
M. Makarenko - maksim.makarenko@kaust.edu.sa,
A. Burguete-Lopez - arturo.burguetelopez@kaust.edu.sa
**Corresponding author: A. Fratalocchi - andrea.fratalocchi@kaust.edu.sa - +966128080348*

SUPPLEMENTARY NOTE I

The activation function $\boldsymbol{\sigma} = \frac{\mathbf{K}}{i(\omega-\boldsymbol{\Omega})+\boldsymbol{\Gamma}}$ is obtained from the solution of the following differential equation:

$$\frac{d\tilde{\boldsymbol{\sigma}}}{dt} = (i\boldsymbol{\Omega} - \boldsymbol{\Gamma})\,\tilde{\boldsymbol{\sigma}} + \mathbf{K}\cdot\delta(t), \tag{1}$$

with $\tilde{\boldsymbol{\sigma}} = \int d\omega \boldsymbol{\sigma}(\omega)e^{i\omega t}$ being the inverse Fourier transform of $\boldsymbol{\sigma}$ and $\delta(t)$ the diract delta function, as the reader can verify by direct substitution. The solution of Eq. (1) reads:

$$\tilde{\boldsymbol{\sigma}}(t) = e^{\mathbf{A}t}\cdot(\tilde{\boldsymbol{\sigma}}_0 + \mathbf{K}), \tag{2}$$

with $\tilde{\boldsymbol{\sigma}}_0 = \tilde{\boldsymbol{\sigma}}(t=0)$ the initial state and $e^{\mathbf{A}t}$ being the matrix exponential, with $\mathbf{A} = i\boldsymbol{\Omega} - \boldsymbol{\Gamma}$. The matrix exponential is expressed in closed form by diagonalizing $\mathbf{A} = \mathbf{Q}\boldsymbol{\Lambda}\mathbf{Q}^{-1}$, with $\Lambda_{mm'} = \lambda_m\delta_{mm'}$ being a diagonal matrix of complex eigenvalues $\lambda_m = i\alpha_m - \beta_m$:

$$e^{\mathbf{A}t} = \mathbf{Q}e^{\boldsymbol{\Lambda}t}\mathbf{Q}^{-1}. \tag{3}$$

In Eq. (3), $\mathbf{Q} = \mathbf{Q}(\boldsymbol{\Omega},\boldsymbol{\Gamma})$ and $\boldsymbol{\Lambda} = \boldsymbol{\Lambda}(\boldsymbol{\Omega},\boldsymbol{\Gamma})$ originate from the eigenvectors and eigenvalues of $\mathbf{A} = i\boldsymbol{\Omega} - \boldsymbol{\Gamma}$ and as such they are functions of $\boldsymbol{\Omega}$ and $\boldsymbol{\Gamma}$. The elements $\tilde{\sigma}_{mn}$ are calculated by substituting (3) into (2):

$$\tilde{\sigma}_{mn}(t) = \sum_h \mu_{mnh}e^{(i\alpha_h - \beta_h)t}, \tag{4}$$

and expressed as the sum of complex damped exponentials with $\mu_{mnh} = \mu_{mnh}(\boldsymbol{\Omega},\boldsymbol{\Gamma})$ coefficients arising from the matrix product of $e^{\mathbf{A}t}\cdot(\tilde{\boldsymbol{\sigma}}_0 + \mathbf{K})$. By applying the Fourier transform on Eq. (4), we obtain the expression of the elements σ_{mn} of $\boldsymbol{\sigma}$:

$$\sigma_{mn}(\omega) = \frac{1}{\sqrt{2\pi}}\sum_h \frac{\mu_{mnh}}{i(\omega-\omega_h)+\gamma_h}, \tag{5}$$

represented as a complex rational function with poles $\omega_h + i\gamma_h$, and coefficients μ_{mnh}.

SUPPLEMENTARY NOTE II

We considered a general rational function:

$$y(\omega) = \frac{\sum_{n=0}^{N_\alpha}\alpha_n\cdot\omega^n}{\sum_{n=0}^{N_\beta}\beta_n\cdot\omega^n}, \tag{6}$$

with $N_\alpha + 1$ and $N_\beta + 1$ coefficients α_n and β_n, respectively. Without loss of generality and for the sake of simplicity, we assumed that the function y_{out} is real. If we control $N_\alpha + N_\beta + 2$ coefficients α_n and β_n, we can then can uniquely determine the behavior of the output function $y_{out}(\omega_m)$ at a set of $m = 1, ..., M = N_\alpha + N_\beta + 1$ spectral frequencies ω_m. To demonstrate this result, we impose the conditions $y_{out}(\omega_m) = y_m(\omega_m)$ on Eq. (6) and obtain the following algebraic system:

$$\left[\mathrm{diag}\left(y(\omega_1),...,y(\omega_M)\right)\cdot V_{N_\beta+1}\ \ V_{N_\alpha+1}\right]\begin{bmatrix}\boldsymbol{\beta}\\ -\boldsymbol{\alpha}\end{bmatrix} = 0, \tag{7}$$

with diag being the diagonal matrix for the M function values $y(\omega_m)$ at the target points, V_i the first i columns of the Vandermonde matrix V generated at the sample points ω_m with components $V_{ij} = \omega_i^{j-1}$, $\boldsymbol{\alpha} = [\alpha_0, ..., \alpha_{N_\alpha}]$ and $\boldsymbol{\beta} = [\beta_0, ..., \beta_{N_\beta}]$ being the rational coefficients sought. The solution to Eq. (6) requires one to find a null vector of the $M \times (N_\alpha + N_\beta + 2)$ matrix:

$$\left[\mathrm{diag}\left(y(\omega_1),...,y(\omega_M)\right)\cdot V_{N_\beta+1}\ \ V_{N_\alpha+1}\right]. \tag{8}$$

For $M = N_\alpha + N_\beta + 1$, the matrix (8) has a null space of dimension $N_\alpha + N_\beta + 2 - M = 1$, and each null vector gives an exact solution in (6) [1]. For $M < N_\alpha + N_\beta + 1$, the system is under determined and the null space of (8) has dimension $N_\alpha + N_\beta + 2 - M > 1$. In this case, any null vector among the space of $\infty^{N_\alpha+N_\beta+2-M}$ provides an exact solution to the problem.

For $M > N_\alpha + N_\beta + 1$, the system is over determined and the solution to Eqs. (6) can be found in the least square sense by constrained minimization:

$$\text{Minimize} \left\| \left[\text{diag}\left(y(\omega_1), ..., y(\omega_M)\right) \cdot V_{N_\beta+1} \quad V_{N_\alpha+1} \right] \begin{bmatrix} \boldsymbol{\beta} \\ -\boldsymbol{\alpha} \end{bmatrix} \right\|, \qquad \text{with} \quad \|\boldsymbol{\alpha}\| + \|\boldsymbol{\beta}\| = 1, \tag{9}$$

with $\|.\|$ being the vector norm. The constraint $\|\boldsymbol{\alpha}\| + \|\boldsymbol{\beta}\| = 1$ is introduced to avoid the trivial solution $\boldsymbol{\beta} = \boldsymbol{\alpha} = 0$.
In the case of Eq. (1) of the main text, the transfer function H is written as follows:

$$H(\omega) = \frac{s_{out}}{s_{in}} = \left[..., \frac{s_{-n}}{s_{in}}, ..., \frac{s_{+n}}{s_{in}} \right] = C(\omega) - \beta\sigma. \tag{10}$$

We begin by considering the case $C(\omega) = C$ independent of the frequency ω. In this case, Eq. (5) shows that H is a complex rational function with coefficients depending on the resonance frequencies Ω and mode dampings Γ. The control of $2M$ resonances Ω_n implies the control of $2M$ coefficients in the transfer function H. Equation (7) shows that we can therefore control the system response in amplitude and phase exactly at $M-1$ spectral points in both real and imaginary part, or equivalently, amplitude and phase in a single scattering channel. At any number of points $\geq M$, the problem can be solved in the least square sense via (9).
This result applies also in the case where $C(\omega)$ is frequency dependent. In this case every condition $H(\omega_l) = H_l$ generates a rational function with different constants $C_l = C(\omega_l)$, maintaining the validity of Eqs. (7)-(9).

SUPPLEMENTARY NOTE III

We considered an optical network with $N = 10$ output scattering channels, initialized with random scattering $C = e^{iH}$, with H being a random symmetric matrix with elements drawn from a uniform distribution between zero and 2π. The random couplings $K = A + iB$ are initialized with random matrices A and B, each with elements drawn from a uniform distribution. We then initialized the dampings to $\Gamma = \frac{KK^\dagger}{2}$. The transmission T is measured in the first channel s_{+1} computed at $n = 1$.

SUPPLEMENTARY NOTE IV

We use the generalized cost function defined in Eq. (6) of the main text to design an 800 nm robust ($\alpha = 0.5$) polarizer, and compare it to a design found without taking into account tolerance to the fabrication errors ($\alpha = 0$). To model fabrication tolerances we varied the sizes of individual boxes using a uniform distribution with random values in the range ± 25 nm. The main contribution to the errors being the proximity effect at the electron beam lithography stage, which affects the sizes but not the position of the centers of the boxes, nor the overall period.

[1] D. Braess, *Nonlinear Approximation Theory* (Springer-Verlag, Berlin, Heidelberg, 1986).

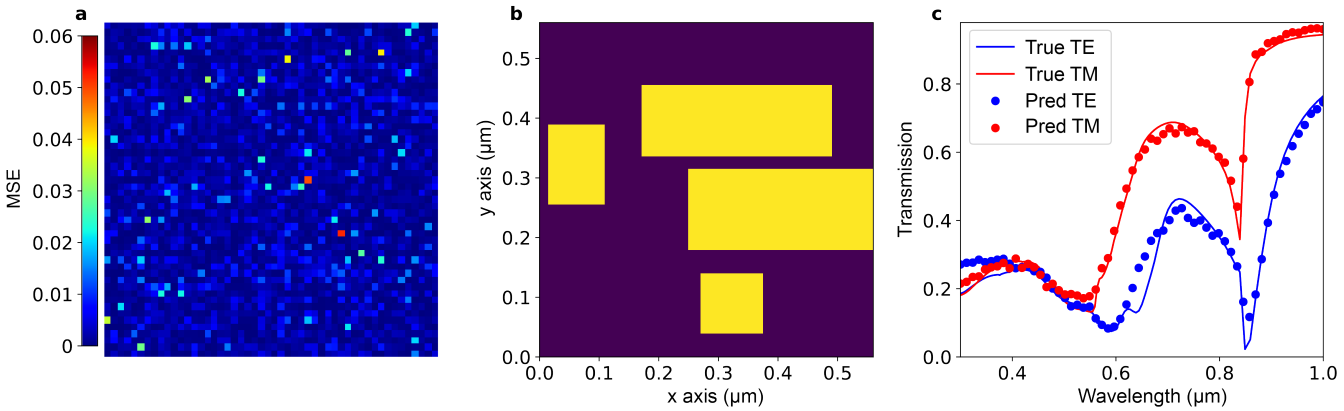

Supplementary Figure 1. ALFRED performance on 2500 randomly chosen samples from a test dataset **a**. The colors represent the mean squared error between the ground truth and the predicted spectra for each sample. An example of the output from the predictor part of ALFRED for the random test sample **c**. The blue and red curves on the figure represent TE and TM spectra respectively, for the true values and dotted lines represents neural network predictions. Figure **b** represents a masked array structure for the given sample.

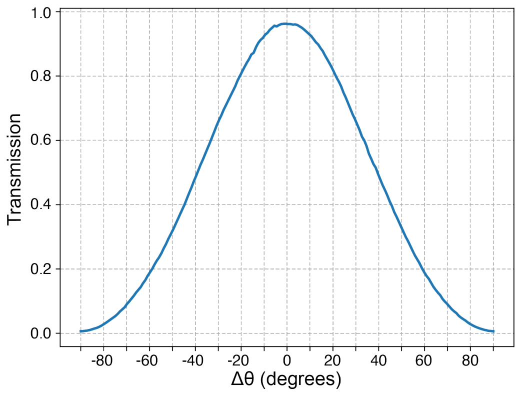

Supplementary Figure 2. Transmission efficiency of 600 nm polarizer at the designed wavelength of 600 nm wavelength versus input polarization angles $\Delta\theta$.

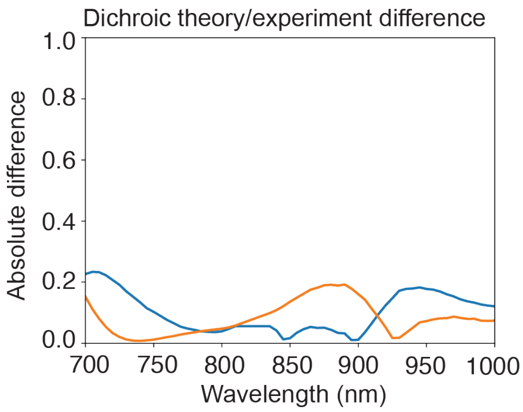

Supplementary Figure 3. Absolute difference between theoretical and experimental transmission curves for the dichroic mirror of Fig. 5 of the main text.

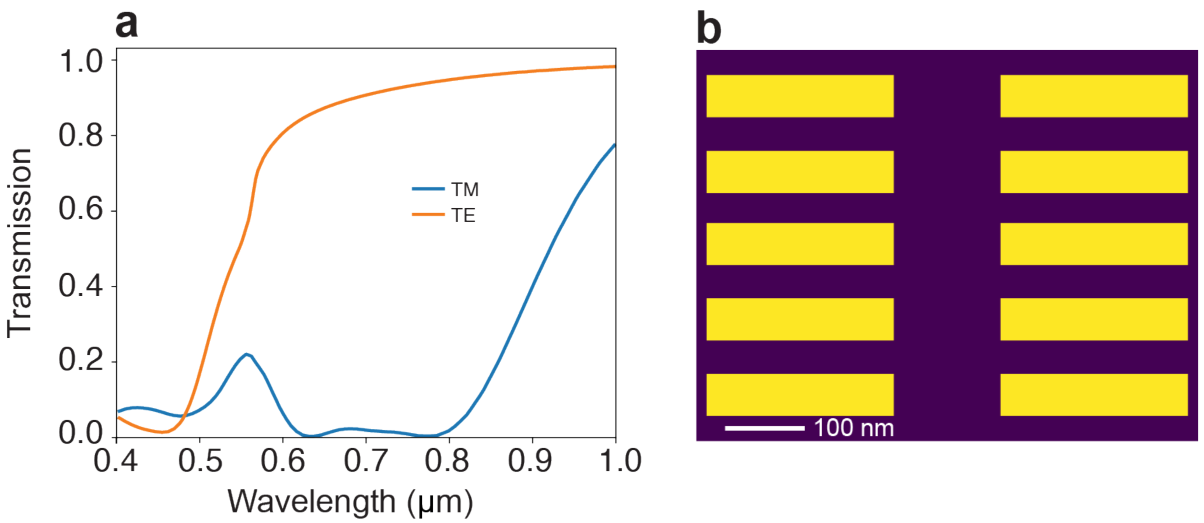

Supplementary Figure 4. Broadband polarizer theoretical transmission curves (Panel **a**) and its masked array structure (Panel **b**). The integral of the difference between TE and TM in the interval from 0.6 to 0.8μm was used as the cost function.

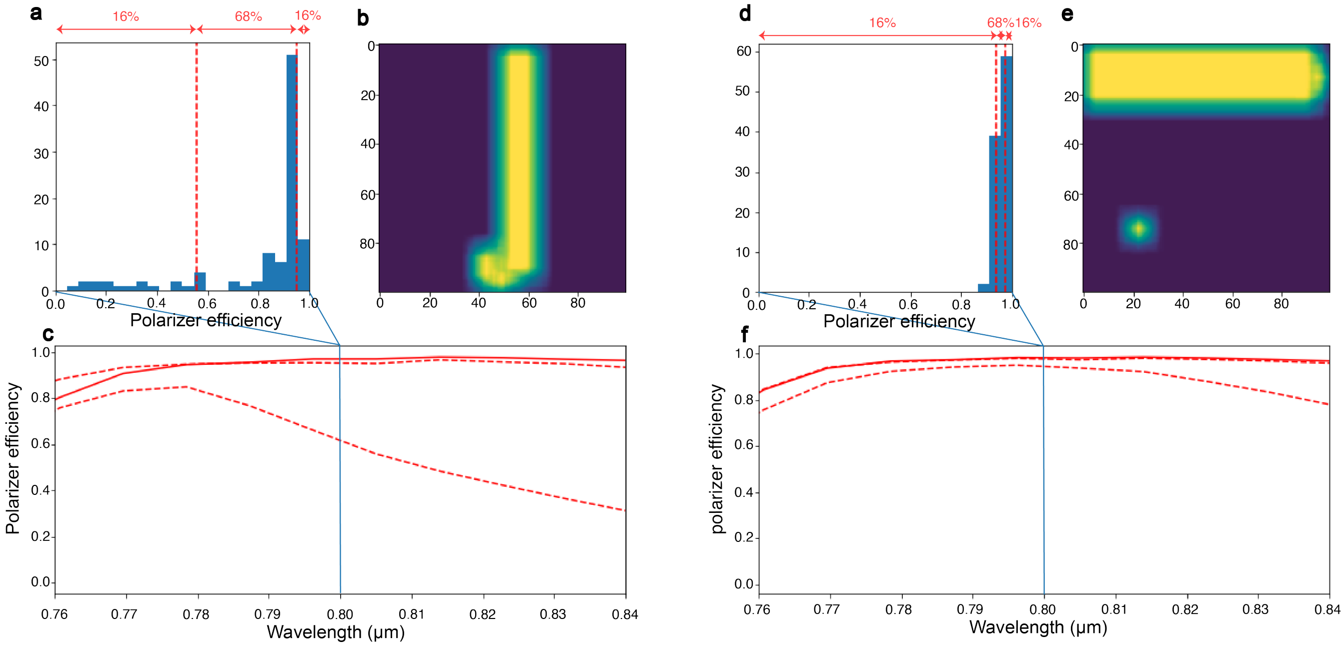

Supplementary Figure 5. Panels **a**-**c** shows results of a 800 nm polarizer designed $\alpha = 0$, while **d**-**f** present results for $\alpha = 0.5$. The solid red line in Panels **c** and **f** represents the efficiency of the polarizer with no random perturbation. The histograms in panels **a** and **d** report the distribution of the whole set of perturbed polarizers. As the probability distribution is not perfectly Gaussian, we split the probability it into a bottom, middle and a top region comprising 16%, 68%, and 100% − 16%, respectively, of the total probability area. The central 68% of all values is chosen to be equivalent to one standard deviation of a normal distribution. The dotted lines in panel **c** shows the minimum efficiency of the polarizers comprising the set at 68%, implying that 68% of the polarizer designs have efficiency higher than that the dotted limit. Panels **c** and **f** reports the same analysis for $\alpha = 0.5$. Panels **b** and **e** report the overlay of all perturbed structures used in this analysis, respectfully for $\alpha = 0$ and $\alpha = 0.5$.
